# Procrastination, stress, and self‐rated health: A multi‐sample meta‐analysis

**DOI:** 10.1111/bjhp.70099

**Published:** 2026-08-08

**Authors:** Fuschia M. Sirois, Cormac Monaghan

**Affiliations:** ^1^ Department of Psychology Durham University Durham UK; ^2^ Hamilton Institute Maynooth University Kildare Ireland

**Keywords:** health status, procrastination, self‐rated health, stress

## Abstract

**Objectives:**

Despite a growing evidence base indicating that trait procrastination increases risk for specific outcomes reflecting poor health, there is less evidence examining the implications of procrastination for overall health status, or investigating the contributing factors involved. Guided by the cognitive process model of self‐rated health (SRH), and the procrastination‐health model, the current study extended previous research and theory by quantifying the link between trait procrastination and SRH across multiple samples and testing the contributions of stress. Additionally, we explored the link between chronic procrastination and the SRH–future SRH discrepancy.

**Methods:**

Thirty‐six cross‐sectional samples (*N* = 8603) completed measures of trait procrastination, current and future SRH. A subset completed measures of perceived stress. Random effects meta‐analyses were conducted on the raw and semi‐partial correlations of procrastination with SRH and FSRH, controlling for perceived stress. Moderator analyses were conducted where warranted. Procrastination scores were examined in relation to the discrepancy between current and future SRH.

**Results:**

Trait procrastination was significantly associated with poor SRH (*r*_avg_ = .21; 95% CIs [.19, .23]), and to a lesser extent, poor future SRH (*r*_avg_ = .13; 95% CIs [.09, .18]). The semi‐partial effects were reduced but significant for SRH, and non‐significant for future SRH. Procrastination was modestly associated with the discrepancy between current and future SRH, reflecting an expectation that health would improve slightly over the next 10 years.

**Conclusions:**

The current findings indicate that chronic procrastination is associated with poor overall health status that can be explained in part by higher levels of stress.

**Statement of Contribution**

**What is already known on this subject?**

- Trait procrastination is associated with specific health‐related outcomes.
- Theory suggests that stress is one route that links procrastination to poor health.
- Research into how and why procrastination may be linked to overall health status, including self‐rated health (SRH), is limited.

**What does this study add?**

- Procrastination is associated with poor current and future SRH across multiple samples.
- Higher perceived stress explains this association in part.
- Procrastination is linked to appraising future SRH as being better than current SRH.

## INTRODUCTION

Trait procrastination is a form of chronic self‐regulation failure involving the unnecessary and voluntary delay of important intended tasks despite knowing that negative consequences may result (Klingsieck, 2013; Sirois & Pychyl, 2013), that is increasingly being recognized as a risk factor for poor health. A growing evidence base has highlighted that chronic procrastination confers risk for a range of adverse health outcomes, including poor health behaviours, higher stress, poor sleep quality, acute health problems (Johansson et al., 2023; Kelly & Walton, 2021; Li et al., 2020; Reinecke et al., 2018; Sirois, 2007; Sirois et al., 2015, 2023; Sirois & Biskas, 2024), and even hypertension and cardiovascular disease (Sirois, 2015a). The importance of understanding the health consequences of procrastination and the processes that contribute to this risk is further underscored by its prevalence. Estimates suggest that 50% of the student, and 15%–25% of the adult population chronically procrastinate (Ferrari et al., 2005), with health being the life domain where adults reported the highest levels of procrastination in one study (Hen & Goroshit, 2018).

Despite this burgeoning evidence base demonstrating the health‐related consequences of procrastination, there is little research into whether the links between procrastination and poor health outcomes translate into poor overall health status. Among the various indicators of health status, self‐rated health (SRH) is a robust, summary measure that is known to be a reliable predictor of morbidity and mortality (Jylhä, 2009), and biomarkers predictive of health (Kananen et al., 2021), after accounting for other risk factors. To date, findings from the limited research examining whether chronic procrastination is linked to SRH are inconclusive and have been conducted with specialized samples (Basirimoghadam et al., 2020; Johansson et al., 2023; Sirois & Biskas, 2024). It is also unclear whether stress, a key explanatory route proposed by the procrastination‐health model (Sirois, 2007; Sirois et al., 2003) linking chronic procrastination to poor health, holds relevance for understanding why procrastination might be linked to poor SRH. The current study addressed these gaps and extended theory by investigating across a range of diverse samples the extent to which procrastination is linked to SRH and by extension, future self‐rated health (FSRH), and the potential explanatory role of stress.

### Procrastination and self‐rated health

Investigations into the health consequences of chronic procrastination have tended to focus on specific outcomes rather than overall health status. This is somewhat surprising given the known contributions of health behaviours, stress, sleep quality, and acute health issues to overall health (e.g., Hirotsu et al., 2015; Wirtz & von Känel, 2017), each of which have been identified as a consequence of chronic procrastination (Johansson et al., 2023; Li et al., 2020; Sirois et al., 2015, 2023). To date, three studies have examined the link between procrastination and SRH, a widely used and robust indicator of health status. In two cross‐sectional studies, nurses prone to procrastination reported poorer SRH (Basirimoghadam et al., 2020; Sirois & Biskas, 2024). However, in a longitudinal study of Swedish university students, a tendency to procrastinate was not significantly associated with SRH 9 months later (Johansson et al., 2023). Although it could be argued that the relative health and youth of the student sample compared with that of the nurses in the other studies (e.g., mean age = 41.09; (Sirois & Biskas, 2024)) may account for this null finding, procrastination was predictive of a number of other specific health outcomes in the student sample (Johansson et al., 2023). It therefore remains unclear the extent to which chronic procrastination may contribute to overall health status, and the extent to which this varies across different populations.

**FIGURE 1:**
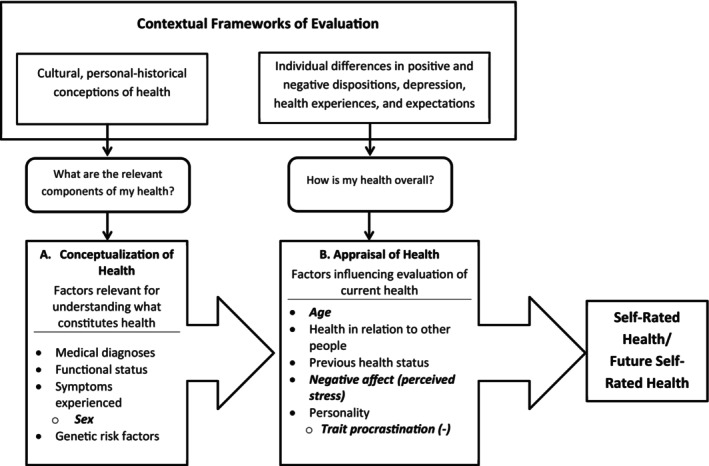
The role of contextual factors in self‐rated health as suggested by the cognitive process model of self‐rated health (Jylhä, 2009) and extended to future self‐rated health. Boxed arrows represent the processes involved in individual health evaluation and not causal pathways. Contributors and/or moderators tested in relation to self‐rated health in the current study are presented in bold, italic font.

From a conceptual perspective, it is reasonable to expect that procrastination impacts SRH. According to the cognitive process model (Jylhä, 2009), SRH arises from an active cognitive process involving both reflective and intuitive evaluations of health that is necessarily contextualized within a framework of individual and socio‐cultural differences. The answer to the question ‘How do you rate your current health?’ is viewed as a product of a multi‐stage process that involves first considering the relevant components of health in terms of cultural and personal‐historical factors, including medical status, symptoms, risk factors, and biological sex (see Figure 1). Health is then appraised through the lens of individual states, traits, and social comparisons. Relevant for the current study, stress and negative affective states can impact how people interpret physical symptoms by drawing attention to physical states (Fischer et al., 2016), and/or eliciting physiological changes that may be viewed as signs of illness (Cohen & Williamson, 1991). As a trait characterized by high levels of negative affect (van Eerde, 2003), chronic procrastination would therefore be expected to contribute to negative evaluations of one's health. Indeed, research applying the cognitive process model found that other traits characterized by negative affect, such as perfectionistic concerns (Sirois & Molnar, 2017), and related to procrastination (Sirois et al., 2017), were associated with poorer SRH (Sirois & Molnar, 2017).

### Procrastination and future self‐rated health

In addition to the appraisals of current health status captured by SRH, there is also some evidence that appraisals of future health may be shaped by personality traits such as chronic procrastination. Across two chronic illness samples, neuroticism predicted variance in a temporal variant of SRH, poor FSRH, over and above demographic variables, fatigue and current SRH (Sirois, 2015b). Similarly, neuroticism predicted steeper declines in FSRH over time in a large population‐based sample (Löckenhoff et al., 2012). Given that neuroticism is a big five personality factor that is moderately linked to trait procrastination (van Eerde, 2003), it is reasonable to expect that chronic procrastination would also be associated with poor FSRH. From the lens of the cognitive process model, people who chronically procrastinate might draw upon their own past experiences with putting off health‐promoting behaviours and incurring additional stress due to procrastination as sources of information to inform their judgements of what their future health may be like, with the result being poor FSRH. Research examining procrastination in relation to health‐related possible selves provides some support for this proposition. In a sample of adults working towards making healthy lifestyle changes, trait procrastination was associated with lower outcome and efficacy expectations for achieving a hoped‐for health‐related possible self (Sirois, 2021).

Whereas SRH reflects actual health states seen through the lens of individual and contextual factors, arguably FSRH reflects expectations for future health states which, although grounded in current health, are also influenced by the individual's own biases with respect to hoped‐for and feared future health states. Procrastination is characterized by avoidant coping, including denial, when dealing with negative states (Sirois & Kitner, 2015), and wishful thinking (Sigall et al., 2000). It is therefore possible that, despite current poor health habits and states, people prone to procrastination may downplay this information when appraising future health and expect that their future health will be better than their current health state. This would be reflected as a larger positive discrepancy between SRH and FSRH. To date, there is little or no research examining trait procrastination in relation to FSRH, and none that we are aware of investigating whether procrastination is linked to a discrepancy between SRH and FSRH.

### Procrastination, stress, and self‐rated health

**FIGURE 2:**
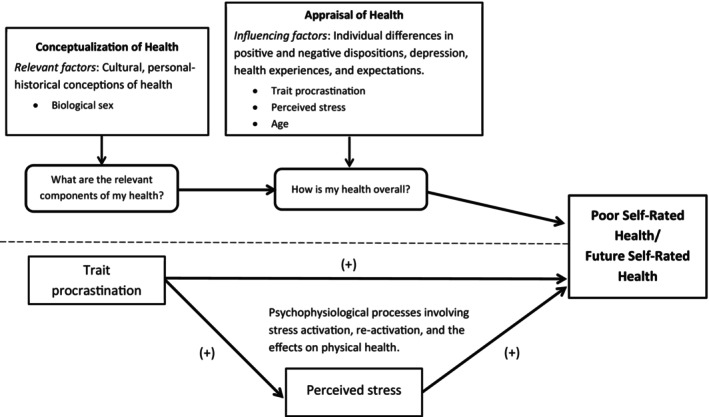
Comparisons of the cognitive process model of self‐rated health (SRH) (Jylhä, 2009), and the procrastination‐health model (Sirois, 2007; Sirois et al., 2003), extended to future SRH, for understanding the roles of trait procrastination and stress in poor SRH. The processes involved in appraisals of health suggested by the cognitive process model (Jylhä, 2009) are presented above the dash line, and the procrastination‐health model (Sirois, 2007; Sirois et al., 2003) is presented below the dashed line. For the cognitive process model, only factors tested in the current study are included in the bullet list.

If, as research and theory suggest, chronic procrastination is associated with poor SRH, understanding and addressing the factors that contribute to this link is important for reducing the health consequences of procrastination. According to the procrastination‐health model (Sirois, 2007; Sirois et al., 2003), stress is one potential explanatory route for understanding why procrastination may confer risk for poor health. Unlike the cognitive process model, which views stress as a contextual factor that can influence cognitive appraisals of health, the procrastination‐health model focuses on the psychophysiological and behavioural processes associated with procrastination that contribute to poor health outcomes. Not only does regular procrastination behaviour generate unnecessary stress (Johansson et al., 2023), but the self‐deprecating and ruminative thoughts about one's own procrastination can both create and maintain stress (Flett et al., 2012; Stainton et al., 2000). Over time, these ruminative and perseverative thoughts can contribute to chronic stress by repeatedly reactivating acute stressors and interfering with healthy adaptation to stress (Smyth et al., 2013). Chronic and/or repeated stress, in turn, creates vulnerability for physical health issues through suppression of the immune system and dysregulation of inflammatory responses (Cohen et al., 2012). From the perspective of the procrastination‐health model (Sirois, 2007; Sirois et al., 2003), poor SRH would therefore be a reflection of the toll that the stress associated with chronic procrastination has on health. In short, although both models highlight stress as a key contributing factor linking chronic procrastination to poor SRH, the processes proposed to explain this link differ between models. Figure 2 provides a model of trait procrastination and SRH that contrasts the processes proposed by each model.

Evidence from both cross‐sectional and longitudinal studies supports the notion that higher stress may contribute to the link between trait procrastination and various health outcomes including poor sleep quality (Sirois et al., 2015), acute health problems (Sirois, 2007; Sirois et al., 2023), and SRH (Sirois & Biskas, 2024). Higher stress is also linked longitudinally to poor SRH (Barry et al., 2021; Svedberg et al., 2006). What remains to be answered is whether stress also contributes to the proposed associations of chronic procrastination with overall current and future SRH, and if so, whether one model might offer a better explanation for this association than the other.

### The current study

Drawing on previous research indicating that trait procrastination increases risk for poor health outcomes, and informed by the cognitive process model of SRH and the procrastination‐health model, the current study aimed to investigate the extent to which trait procrastination is linked to poorer SRH and FSRH. We also tested the potential contribution of perceived stress in this association, with higher stress expected to account in part for the link between trait procrastination and poor SRH. Extending both models, we further explored whether trait procrastination is linked to discrepancies between current and future evaluations of health.

To provide more robust tests of our hypotheses and exploratory analyses, we statistically meta‐analysed data from samples collected from our own labs. This Meta‐analysis of [one's] Own Data or ‘MOD’ approach has several key advantages. First, it can contribute to building a robust evidence base for an understudied area or where the published literature is not sufficient to conduct a traditional meta‐analysis (see Cumming, 2014). Second, the MOD approach enables a systematic investigation of potential moderators. For the current study, we explored the possible impact of demographic (age, participant sex) and methodological (sample type, procrastination scale) moderators on the average effects. Finally, a MOD approach provides the opportunity to address the question of *how* procrastination may impact SRH by testing for the role of higher perceived stress in this association, and meta‐analysing the adjusted effects. Consistent with both the cognitive process model (Jylhä, 2009) and the procrastination‐health model (Sirois, 2007; Sirois et al., 2003), we expected that once the contribution of stress was partialled out of the associations of procrastination and SRH, the magnitude of the associations would be reduced.

## METHODS

### Participants and procedure

This pre‐registered study (https://osf.io/3apfg/overview?view_only=77d2385996ca45f78c956fa0823898d2) analysed data from 36 independent samples (total *N* = 8603), including eight undergraduate and graduate student samples and 28 community adult samples. Data were collected over a 14‐year period (2011–2025) as part of multiple broader research programmes investigating the correlates of personality and health. Ethical approval for all data collection procedures was obtained from the relevant Institutional Research Ethics Boards in Canada and the United Kingdom.

Of the 36 data sets, all but two were collected completely online. In sample 4, 264 of the responses were collected via mail‐in surveys, and in sample 21, 68 responses were collected via mail‐in surveys, and the rest online. Except for sample 4, all samples were convenience samples recruited through the project team's research labs. Sample 4 was recruited from a random sample of 1000 individuals drawn from a nursing association database. All but two samples used a cross‐sectional design. S22 and 30 had a two time point prospective design, but only the baseline data included the variables of interest and was therefore analysed to be consistent with the other data sets.

### Measures

Participants provided standard demographic information, including age, gender, ethnicity, and education level (Table 1). Descriptive statistics and internal consistency estimates (Cronbach's α) for all measures are reported in Table 2.

**TABLE 1:** Demographic characteristics of the 36 samples (total *N* = 8603).

Sample	*N*	Percent female	Percent white	Age (years)		Education level (%)		
				*M*	*SD*	High school	College/university	Postgraduate
S1	296	.76	.69	28.18	11.71	.09	.63	.23
S2	327	.81	.57	21.76	4.94	.00	1.00	.00
S3	70	.87	.89	35.39	14.25	.10	.61	.29
S4	587	.94	.73	40.93	12.73	.00	1.00	.00
S5	327	.73	.75	28.90	12.87	.17	.55	.28
S6	114	.68	.86	37.77	12.94	.04	.42	.54
S7	123	.71	NA	20.70	3.16	.16	.83	.01
S8	101	.81	.65	32.50	15.40	.06	.73	.21
S9	318	1.00	.81	24.28	5.38	.07	.59	.34
S10	318	.78	.81	28.94	10.80	.09	.53	.39
S11	150	.50	NA	42.83	13.77	.17	.64	.19
S12	298	.78	.73	27.36	12.99	.55	.10	.34
S13	87	.75	.89	49.38	8.52	.13	.45	.43
S14	129	.77	.43	30.76	13.42	.00	.88	.12
S15	725	.69	.82	30.51	12.23	.09	.51	.40
S16	111	1.00	.59	26.79	10.68	.09	.83	.08
S17	162	.78	.78	22.18	5.54	.00	1.00	.00
S18	451	.71	.74	23.31	6.70	.13	.61	.25
S19	187	.74	NA	22.50	5.90	.00	1.00	.00
S20	318	.77	.53	29.26	13.73	.00	1.00	.00
S21	170	.74	.54	33.48	17.42	.12	.80	.08
S22	271	.70	.63	21.32	4.44	.00	1.00	.00
S23	305	.76	.80	25.56	12.61	.21	.69	.10
S24	195	.82	.63	19.86	2.14	.32	.65	.03
S25	124	.76	.86	24.35	11.73	.34	.56	.10
S26	163	.69	.74	31.09	13.27	.15	.62	.24
S27	285	.69	NA	25.17	6.32	.10	.57	.32
S28	239	.69	NA	30.18	11.83	.12	.67	.21
S29	266	.78	.56	20.42	3.74	.12	.87	.01
S30	322	.62	.80	36.37	13.95	.13	.60	.27
S31	346	.73	.74	28.85	12.78	.17	.55	.28
S32	106	.74	.45	24.08	4.51	.12	.28	.60
S33	132	.54	.81	21.53	5.33	.07	.93	.00
S34	100	.51	.66	24.50	3.49	.25	.56	.19
S35	100	.50	.97	65.10	4.53	.35	.41	.24
S36	280	.50	.78	39.00	12.30	.20	.56	.23

**TABLE 2:** Summary of the characteristics of the study variables for the 36 independent samples (total *N* = 8603).

Sample (*N*)	Trait procrastination			Stress			Self‐rated health^a^		Future self‐rated health^a^	
	α	*M*	*(SD)*	α	*M*	*(SD)*	*M*	*(SD)*	*M*	*(SD)*
S1 (296)	.90	2.85	.67	–	–	–	2.40	.98	–	–
S2 (327)	.86	2.68	.55	.84	2.96	.55	2.36	.85	–	–
S3 (70)	.92	3.22	.89	.87	3.35	.66	4.03	.95	3.59	1.04
S4 (587)	.81	2.87	.92	.86	2.60	.58	2.41	.86	–	–
S5 (327)	.88	3.14	.75	.85	3.01	.65	2.73	.88	2.34	.90
S6 (114)	.88	3.41	.72	.88	2.90	.67	2.51	.83	2.44	.83
S7 (123)	.86	2.74	.62	.89	2.87	.70	2.41	.92	2.05	.74
S8 (101)	.89	2.94	.81	–	–	–	2.76	1.01	2.36	1.09
S9 (318)	.90	3.11	.79	–	–	–	2.53	.92	2.39	.91
S10 (318)	.90	3.14	.83	.89	2.99	.69	2.79	.96	2.43	.96
S11 (150)	.88	3.27	.83	.92	3.36	.79	3.98	.85	4.01	1.05
S12 (298)	.87	3.06	.74	–	–	–	2.60	.86	2.35	.87
S13 (87)	.89	2.83	.69	–	–	–	2.46	.94	2.66	.76
S14 (129)	.91	2.93	.82	.88	3.00	.69	2.67	.96	2.57	.93
S15 (725)	.90	3.10	.80	–	–	–	2.40	.91	2.40	.92
S16 (111)	.91	2.55	.69	–	–	–	2.80	.92	2.55	1.04
S17 (162)	.87	2.91	.62	.85	2.75	.60	2.25	.86	–	–
S18 (451)	.90	3.15	.77	–	–	–	2.49	.92	2.24	.84
S19 (187)	.88	2.68	.58	.86	2.92	.60	2.20	.80	–	–
S20 (318)	.88	3.04	.76	–	–	–	2.90	.83	2.55	.84
S21 (170)	.88	2.54	.65	.89	2.79	.76	2.36	.88	–	–
S22 (271)	.87	2.70	.61	.87	2.72	.68	2.19	.79	–	–
S23 (305)	.89	3.12	.82	–	–	–	2.26	.94	–	–
S24 (195)	.89	3.31	.79	–	–	–	2.47	.90	–	–
S25 (124)	.91	3.31	.86	–	–	–	2.29	.79	–	–
S26 (163)	.90	3.54	.80	–	–	–	2.67	.96	–	–
S27 (285)	.86	3.38	.73	–	–	–	2.48	.87	–	–
S28 (239)	.82	2.91	.77	–	–	–	2.27	.95	–	–
S29 (266)	.82	3.25	.62	–	–	–	2.32	.96	–	–
S30 (322)	.89	2.80	.84	–	–	–	2.36	.91	–	–
S31 (346)	.88	3.15	.75	–	–	–	2.74	.89	2.36	.89
S32 (106)	.85	3.25	.75	–	–	–	2.44	.83	–	–
S33 (132)	.87	3.06	.73	.86	2.77	.66	2.78	.93	2.20	.94
S34 (100)	.90	3.12	.81	–	–	–	2.57	1.03	2.46	.91
S35 (100)	.90	2.48	.73	–	–	–	2.85	.99	3.52	.90
S36 (280)	.92	2.97	.88	–	–	–	2.57	.98	2.80	1.05

^a^ Note that SRH and FSRH were coded with higher values reflecting poorer health.

#### Trait procrastination

Trait‐level procrastination was assessed using one of three established self‐report instruments: in eight of the samples (samples 1, 2, 7, 16, 17, 19, 21, 22) the General Procrastination Scale (GPS; Lay, 1986), in 27 of the samples (samples 3, 5, 6, 8–15, 18, 20, 23–36) its nine‐item short‐form version (GPS‐9; Sirois et al., 2019), and in sample 4, the Revised Adult Inventory of Procrastination (AIPR; McCown & Johnson, 2001). The GPS includes 20 items (e.g., ‘In preparing for some deadlines, I often waste time by doing other things’) rated on a 5‐point Likert scale ranging from 1 (strongly disagree) to 5 (strongly agree). After reverse‐scoring 10 items, a mean score is calculated, with higher scores indicating a greater general tendency to procrastinate. The GPS‐9 consists of nine items (e.g., ‘I often find myself performing tasks that I had intended to do days before’) from the full GPS, using the same response scale. Three items are reverse scored, and an average score is computed, with higher scores reflecting a stronger tendency to procrastinate. The AIPR includes 15 core items (e.g., ‘I often think that I don't get things done on time’) rated on a 7‐point scale from 1 (strongly disagree) to 7 (strongly agree) and 5 distractor items that are not scored. Scores are averaged, with higher scores indicating stronger procrastination tendencies.

All three scales have demonstrated strong psychometric properties. Previous studies report excellent internal consistency for the GPS (α=.90; Sirois, 2007), good to excellent reliability for the GPS‐9 (α=.85; Manap et al., 2024), and similarly strong reliability for the AIPR (α=.84; McCown & Johnson, 2001). In the present study, reliability estimates were also high: GPS‐20 ($\alpha \in \left[.86-.92\right]$), GPS‐9 $\left(\alpha \in \left[.81-.92\right]\right)$, and AIPR $\left(\alpha =.81\right)$.

#### Perceived stress

In 14 of the samples (samples 2–7, 10, 11, 14, 17, 19, 21, 22, and 33) perceived stress was assessed using the Perceived Stress Scale (PSS‐10; Cohen et al., 1983), a 10‐item measure designed to capture individuals' appraisal of stress experienced over the past month. Participants rated each item (e.g., ‘In the last month, how often have you felt that you were unable to control the important things in your life?’) on a 5‐point scale ranging from 0 (never) to 4 (very often). Items reflecting positive experiences or coping (e.g., ‘In the last month, how often have you felt confident about your ability to handle your personal problems?’) are reverse‐coded, and a mean score calculated, with higher scores reflecting greater perceived stress. A prior review of the PSS‐10's psychometric properties across 12 studies reported good to excellent internal consistency ($\alpha \in \left[.74-.91\right]$; Lee, 2012). In the current study, reliability was similarly strong, ranging from good (α=.81; sample 33) to excellent (α=.91; sample 11).

#### Self‐rated health

Current SRH was assessed with the widely used single‐item measure of general health from the Medical Outcomes Survey 36 item short‐form (SF‐36) health questionnaire (Ware & Sherbourne, 1992). Single‐item SRH measures are frequently used in health research due to their strong predictive validity for a range of outcomes (Shooshtari et al., 2007). In all samples (except sample 16), respondents rated their current health on a 5‐point scale ranging from 1 (excellent) to 5 (poor), such that higher scores reflected poorer SRH. In Sample 16, a continuous measure was used ranging from 0 (poor) to 100 (excellent).

Future self‐rated health (FSRH) was assessed in 20 of the samples (samples 3, 5–16, 18, 20, 31, and 33–36) using a parallel item that asked participants to anticipate the state of their physical health 10 years in the future. As with the current SRH measure, all but one sample used the same 5‐point scale ranging from 1 (excellent) to 5 (poor), with sample 16 rating expectations for future health rated on the continuous 0–100 scale.

Across all datasets, SRH and FSRH scores were coded such that higher values indicated poorer health. Where necessary, scales were reverse‐coded prior to analysis to ensure a consistent interpretation across studies.

### Data analysis

All data analysis was carried out using a combination of R (version 5.0; Team, 2025) and SPSS (version 29.0). Pearson's correlation coefficients were computed separately for both SRH and FSRH. Specifically, separate correlations were calculated to examine the bivariate associations between trait procrastination and both SRH and FSRH (*r*_y1_), between trait procrastination and perceived stress (*r*_12_), and between perceived stress and SRH and FSRH (*r*_y2_). Following this, because we were interested in the unique contribution of trait procrastination to SRH and FSRH (*sr*_1_), separate semi‐partial correlations controlling for perceived stress were computed for each form of SRH.

All r values were interpreted in line with Cohen's (1988) guidelines, where r=.10 is considered a small‐sized effect, r=.30 is considered a medium‐sized effect, and r=.50 is considered a large‐sized effect. The resulting adjusted correlations were subsequently included in meta‐analytic models.

To estimate the average association between trait procrastination and both SRH and FSRH across studies (k=36 for SRH; k=20 for FSRH) random‐effects meta‐analyses were conducted using the Comprehensive Meta‐analysis (CMA; Version 4; Borenstein et al., 2022) software. Prior to aggregation, CMA transforms the individual correlation coefficients $\left(r\right)$ into Fisher's *z*‐scores.

#### Heterogeneity and moderator analyses

To evaluate heterogeneity across effect sizes and determine the need for moderator analyses, two metrics were used. First, the Q statistic tested for the presence of heterogeneity across studies; a significant Q and wide confidence intervals suggested variability warranting further investigation. Second, the $I^{2}$ statistic estimated the proportion of variance attributable to between‐study heterogeneity rather than sampling error. Values of $I^{2}=25\%,50\%,\text{and}\;75\%$ were interpreted as indicators of low, moderate, and high heterogeneity, respectively.

Moderator analyses were conducted to examine the potential influence of both demographic (age, percentage female) and methodological (sample type; GPS scale version) variables on the observed associations and semi‐partial effects. Subgroup analyses were only conducted when there were three or more studies per subgroup, following the recommendations of Card (2012) to avoid reduced statistical power and inflated risk of spurious findings in small subgroups.

Moderator analyses followed a mixed‐effects modelling approach. A random‐effects model was used to analyse the combined subgroups and to estimate within‐group heterogeneity. A fixed‐effects model was then used to assess heterogeneity between subgroups. For continuous moderators (age, percentage female), mixed‐effects meta‐regressions using the method of moments were conducted. Meta‐regression analyses were only performed when there were 10 or more studies in the dataset, in line with established recommendations for ensuring sufficient statistical power.

#### Robustness checks

The robustness of the meta‐analytic findings was evaluated using Rosenthal's (1979) Fail‐safe *N*, which estimates the number of unpublished or null‐result studies that would be required to reduce the overall effect to non‐significance. Of the 36 datasets included in the meta‐analyses, 21 were unpublished. Nonetheless, the Fail‐safe *N* was still calculated given that all datasets originated from the authors' lab. Following Rosenthal (1979) guidelines, the Fail‐safe *N* was considered adequate if it exceeded the threshold of 5*k* + 10, where *k* is the number of studies included in the analysis.

#### Exploratory analysis: Self‐rated health discrepancy

In line with our pre‐registered exploratory question, we examined whether trait procrastination was associated with the discrepancy between participants' current SRH and their anticipated FSRH (see Figure S1 for an illustration of the discrepancy). For each dataset, a discrepancy score was calculated as (SRH – FSRH) + 5. The constant 5 was added so that a value of 5 represented no discrepancy between SRH and FSRH (SRH = FSRH). Because SRH and FSRH were coded with higher values reflecting poorer health, values above 5 indicated that participants expected their future health to be better than their current health (SRH > FSRH), whereas values below 5 indicated expectations of poorer future health relative to current health (SRH < FSRH). Following this, we evaluated the relationship between trait procrastination and this discrepancy via a regression model fitted to each dataset. In addition, to examine whether any observed associations were accounted for by age differences, we conducted a second set of regressions that included both procrastination and age as predictors.

Finally, to synthesize effects across studies, regression coefficients (β values for procrastination) were extracted and subjected to random‐effects meta‐analysis in R using the *metafor* package (Viechtbauer, 2010). This was done both for the simple models (procrastination only) and the age‐controlled models. The comparison of these two sets of results allowed us to evaluate whether the observed links between procrastination and the SRH‐FSRH discrepancy remained after adjusting for age, or whether they were largely explained by the age‐related variance.

## RESULTS

Of the 36 datasets, 28 contained missing values on key variables and were assessed for patterns of missingness using Little's (1988) MCAR test from the *naniar* package (Tierney & Cook, 2023). Following the recommendations of Jakobsen et al. (2017), datasets with a non‐significant MCAR test (*k* = 18) were imputed using linear interpolation from the *zoo* package (Zeileis & Grothendieck, 2005). For datasets where the MCAR test was significant (*k* = 10), missing values were addressed using multiple imputation with chained equations from the *mice* package (van Buuren & Groothuis‐Oudshoorn, 2011).

### Trait procrastination and SRH

A meta‐analysis of unadjusted correlations across the 36 samples (total *N* = 8603) revealed a significant, positive, medium‐sized association between procrastination and poor SRH (see Table 3). Tests of heterogeneity indicated no significant unexplained variability among effect sizes $Q\left(35\right)=43.53,p=.155,I^{2}=19.45\%$, indicating that moderator analyses were not warranted.

**TABLE 3:** Meta‐analysed effect sizes between trait procrastination (PRO), poor self‐rated health (SRH), and perceived stress across 36 samples (total *N* = 8603).

Sample	*N*	Sample type	Procrastination measure	PRO–SRH *r*	PRO–stress *r*	Stress–SRH *r*	PRO–SRH *Sr*_Stress_
S1	296	Community	GPS 20	.28	–	–	–
S2	327	Student	GPS 20	.25	.31	.29	.18
S3	70	Community	GPS 9	.22	.60	.32	.04
S4	587	Community	AIPR	.22	.40	.33	.10
S5	327	Community	GPS 9	.23	.40	.32	.12
S6	114	Community	GPS 9	.10	.40	.43	−.08
S7	123	Student	GPS 20	.36	.57	.32	.23
S8	101	Community	GPS 9	.10	–	–	–
S9	318	Community	GPS 9	.20	–	–	–
S10	318	Community	GPS 9	.21	.42	.40	.05
S11	150	Community	GPS 9	.22	.41	.45	.05
S12	298	Community	GPS 9	.20	–	–	–
S13	87	Community	GPS 9	.02	–	–	–
S14	129	Community	GPS 9	.15	.29	.29	.07
S15	725	Community	GPS 9	.18	–	–	–
S16	111	Community	GPS 20	.10	–	–	–
S17	162	Student	GPS 20	.06	.24	.12	.03
S18	451	Student	GPS 9	.26	–	–	–
S19	187	Student	GPS 20	.18	.35	.29	.09
S20	318	Community	GPS 9	.29	–	–	–
S21	170	Community	GPS 20	.10	.32	.39	−.03
S22	271	Student	GPS 20	.18	.27	.37	.09
S23	305	Community	GPS 9	.23	–	–	–
S24	195	Student	GPS 9	.26	–	–	–
S25	124	Community	GPS 9	.24	–	–	–
S26	163	Community	GPS 9	.14	–	–	–
S27	285	Community	GPS 9	.11	–	–	–
S28	239	Community	GPS 9	.26	–	–	–
S29	266	Community	GPS 9	.18	–	–	–
S30	322	Community	GPS 9	.14	–	–	–
S31	346	Community	GPS 9	.23	–	–	–
S32	106	Community	GPS 9	.16	–	–	–
S33	132	Student	GPS 9	.15	.42	.27	.04
S34	100	Community	GPS 9	.40	–	–	–
S35	100	Community	GPS 9	.23	–	–	–
S36	280	Community	GPS 9	.37			
Meta‐analysis results							
Average *r* (*k*)				.21	.38	.33	.08
95% CI				[.19, .23]	[.33, .43]	[.29, .37]	[.05, .12]
*N*				8603	3067	3067	3067

*Note*: AIPR = Revised Adult Inventory of Procrastination (McCown & Johnson, 2001); GPS 20 = General procrastination scale (Lay, 1986); GPS‐9 = Nine‐item GPS (Sirois et al., 2019); Note that SRH was coded with higher values reflecting poorer health.

### Trait procrastination, perceived stress, and SRH

The meta‐analysis of associations between procrastination and perceived stress across 14 samples revealed the expected positive relationship (see Table 3). Heterogeneity tests indicated moderate variability among effect sizes, $Q\left(13\right)=27.21,p=.012,I^{2}=52.22\%$. For perceived stress and poor SRH, consistent positive associations were observed across the same 14 samples, with an overall medium‐sized effect (see Table 3). Heterogeneity was non‐significant, $Q\left(13\right)=17.60,p=.173,I^{2}=26.14\%$, and moderator analyses were therefore omitted.

A meta‐analysis of semi‐partial correlations (adjusted for perceived stress) revealed a small but significant positive association between procrastination and poor SRH (see Table 3). Similar to the unadjusted analyses, there was no evidence of heterogeneity, $Q\left(13\right)=12.99,p=.449,I^{2}=0\%$. Compared to the medium‐sized unadjusted effects, the adjusted effects were reduced in magnitude, suggesting that perceived stress partially accounted for the procrastination – poor SRH association.

### Trait procrastination and FSRH

A meta‐analysis of unadjusted correlations across the 20 samples (total *N* = 4598) revealed a significant, positive, small‐sized association between procrastination and poor FSRH (see Table 4). Tests of heterogeneity indicated there was significant unexplained variability among effect sizes $Q\left(19\right)=37.55,p=.007,I^{2}=49.41\%$, suggesting that moderator analyses were warranted.

**TABLE 4:** Meta‐analysed effect sizes between trait procrastination (PRO), poor future self‐rated health (FSRH), and perceived stress across 20 samples (total *N* = 4598).

Sample	*N*	Sample type	Procrastination measure	PRO–FSRH *r*	PRO–stress *r*	Stress–FSRH *r*	PRO–FSRH *Sr*_Stress_
S3	70	Community	GPS 9	.30	.60	.46	.03
S5	327	Community	GPS 9	.10	.40	.16	.04
S6	114	Community	GPS 9	.12	.40	.35	−.02
S7	123	Student	GPS 20	.15	.57	.13	.10
S8	101	Community	GPS 9	.01	–	–	–
S9	318	Community	GPS 9	.10	–	–	–
S10	318	Community	GPS 9	.05	.42	.23	−.05
S11	150	Community	GPS 9	.19	.41	.32	.07
S12	298	Community	GPS 9	.12	–	–	–
S13	87	Community	GPS 9	−.22	–	–	–
S14	129	Community	GPS 9	.10	.29	.08	.08
S15	725	Community	GPS 9	.09	–	–	–
S16	111	Community	GPS 20	.09	–	–	–
S18	451	Student	GPS 9	.21	–	–	–
S20	318	Community	GPS 9	.11	–	–	–
S31	346	Community	GPS 9	.10	–	–	–
S33	132	Student	GPS 9	.15	.42	.19	.08
S34	100	Community	GPS 9	.38	–	–	–
S35	100	Community	GPS 9	.24	–	–	–
S36	280	Community	GPS 9	.28			
Meta‐analysis results							
Average *r* (*k*)				.13	.43	.22	.03
95% CI				[.09, .18]	[.37, .49]	[.167, .27]	[−.02, .08]
*N*				4598	1363	1363	1363

*Note*: GPS 20 = General procrastination scale (Lay, 1986); GPS‐9 = Nine‐item GPS (Sirois et al., 2019); Note that FSRH was coded with higher values reflecting poorer future health.

Moderator analyses for the possible impact of GPS scale version and sample type could not be conducted as there were fewer than 3 studies per subgroup for each of these analyses. The meta‐regression testing the potential influence of participant age on the unadjusted effects for trait procrastination and poor FSRH was non‐significant, *b* = −.00 [.00, −.01], *Q*_model_ (1) = .01, *p* = .90, *Q*_residual_ (18) = 37.55, *p* = .004. However, the meta‐regression examining the influence of participant sex on the associations of trait procrastination with poor FSRH were significant, *b* = −.36 [−.65, −.08], *Q*_model_ (1) = 6.32, *p* = .01, *Q*_residual_ (18) = 28.41, *p* = .06, analogue *R* ^2^ = .41. This indicated that as the proportion of males in the sample decreased, the magnitude of the associations between trait procrastination and poor FSRH increased.

### Trait procrastination, perceived stress, and FSRH

The meta‐analysis of associations between procrastination and perceived stress across eight samples revealed the expected positive relationship (see Table 4). Heterogeneity tests indicated no variability among effect sizes, $Q\left(7\right)=11.79,p=.108,I^{2}=40.62\%$. For perceived stress and poor SRH, consistent positive associations were observed across the same 14 samples, with an overall small to medium‐sized effect (see Table 4). Again, heterogeneity was non‐significant, $Q\left(7\right)=14.01,p=.051,I^{2}=50.04\%$.

Lastly, a meta‐analysis of the semi‐partial correlations (adjusted for perceived stress) revealed a small but non‐significant positive association between procrastination and poor FSRH (see Table 4). Similar to the unadjusted analyses, there was no evidence of heterogeneity, $Q\left(7\right)=3.80,p=.802,I^{2}=0\%$.

### Procrastination and discrepancy between SRH and FSRH

An exploratory analysis of discrepancy between current and future SRH across 20 of the 36 samples ($\text{mean discrepency}\in \left[4.33-5.58\right]$; Figure S1) revealed that the association with trait procrastination was inconsistent across samples. In nine of the samples (samples 5, 7, 9, 10, 13, 15, 18, 20, and 31), higher procrastination scores were significantly associated with a larger gap between current and future SRH, with small‐to‐moderately sized beta values $\left(\beta \in \left[.09-.35\right]\right)$. These results suggest that in some samples, individuals with higher levels of procrastination tended to anticipate greater improvements in their future health relative to their current health. However, several studies showed no reliable effect, with some showing non‐significant negative effects (e.g., sample 3: β=−.11,p=.322), and others having non‐significant positive effects (e.g., sample 34: β=.07,p=.425). This pattern indicates there is substantial variability in the observed associations across independent samples.

**FIGURE 3:**
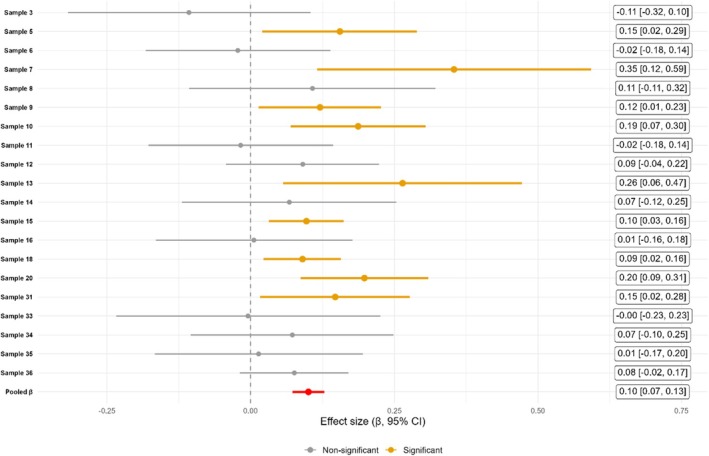
Forest plot of study‐specific and pooled meta‐analytic estimates (β, 95% CI) for the link between procrastination and current– future self‐rated health discrepancies. Note that SRH and FSRH were coded with higher values reflecting poorer health.

To synthesize these findings and test whether this variability was significant, we conducted a random effects meta‐analysis pooling the 20 available samples (Figure 3). The overall effect was small but statistically significant $\left(\beta_{\text{pooled}}=.10\;\left[.07-.13\right];p<.001\right)$, with no evidence of between‐study heterogeneity $\left(Q\left(19\right)=24.50,p=.179,I^{2}=13.0\%\right)$. Taken together, these exploratory findings provide preliminary evidence that trait procrastination may sometimes be linked to overly optimistic expectations about future health, although the effect is modest in size.

In addition to the simple regression models, we conducted a second set of analyses controlling for age to assess whether the association between trait procrastination and the SRH‐FSRH discrepancy was independent of age‐related differences. Across the 20 samples, the inclusion of age as a covariate attenuated some of the previously observed effects, although several significant associations remained. In five of the samples (samples 7, 10, 13, 18, and 20) higher procrastination scores were significantly associated with a larger gap between current and future SRH, with small‐to‐moderately sized beta values (β∈ [.09–.34]).

Again, to synthesize these adjusted effects, we pooled the regression coefficients for procrastination (controlling for age) using a random effects meta‐analysis for the 20 available samples (Figure S2). The overall adjusted effect was smaller in size but remained statistically significant $\left(\beta_{\text{pooled}}=.07\;\left[.04-.10\right];p<.001\right)$. Consistent with the unadjusted models, there was no evidence of between‐study heterogeneity $\left(Q\left(19\right)=23.06,p=.235,I^{2}=.1\%\right)$.

### Robustness checks

The Fail‐safe *N* analysis for the unadjusted correlations between trait procrastination and poor SRH found that to reduce the *p* value below .05 and render the average effect non‐significant, an additional 3262 studies with non‐significant results would need to be included in the set of studies that were statistically meta‐analysed. This value was well above the 5*k* + 10 studies cutoff (190) recommended by Rosenthal (1979).

Because one sample used a measure of trait procrastination that was not a version of the GPS, a sensitivity analysis was conducted for the effects in the overall meta‐analysis of the unadjusted effects. When Sample 4, which used the AIPR, was removed, the overall average effect remained the same, $r_{\mathrm{avg}}=.21,95\%\mathrm{CI}\;\left[.91-.23\right],Q\left(34\right)=43.40,p=.129,I^{2}=21.66\%$, indicating that this variation in procrastination measurement did not significantly impact the overall magnitude of the average association between procrastination and poor SRH.

## DISCUSSION

The current study replicated and extended previous research and theory by quantifying the link between trait procrastination and poor SRH through a robust analysis of 36 diverse samples. Crucially, we also applied the cognitive process model of SRH and the procrastination‐health model to test the potential contribution of stress to the procrastination–SRH link and provide insights into the potential processes involved. Across all samples, procrastination was associated with poor SRH, with a small to moderate but significant average association and a non‐significant degree of variability. Consistent with our hypothesis and both models, when the contributions of perceived stress were accounted for, the magnitude of this association was noticeably attenuated but remained significant. Lastly, the analysis of a subset of 20 samples found that procrastination was significantly associated with poor future SRH, although the average association was small in magnitude. These associations were found to vary significantly, with the magnitude of the associations increasing as the proportion of males in the sample increased. Similar to the analysis of the adjusted associations between procrastination and poor SRH, when stress was accounted for, the magnitude of the average association decreased and was no longer significant. Lastly, the analysis of procrastination and the discrepancy between current and future SRH revealed that those prone to procrastination held expectations that compared with their current health, their future health would improve slightly over the next 10 years.

The current findings contribute to and extend a growing evidence base that underscores the negative health consequences of chronic procrastination in several important ways. Previous research on the risks of procrastination for health has tended to focus on specific aspects of health, including health behaviours, sleep, stress, physical symptoms, and illness (Johansson et al., 2023; Kelly & Walton, 2021; Li et al., 2020; Sirois, 2015a; Sirois et al., 2015, 2023), rather than overall health. Using SRH as a measure of health status, we found evidence that procrastination is also linked to poor overall health, and not just specific aspects. This makes sense if we consider that SRH is a robust summary measure of objective health indicators and outcomes (Jylhä, 2009), including key biomarkers for health (Kananen et al., 2021). Although previous research linking procrastination to SRH is scant and inconclusive, the results of our analysis of 36 samples provide a clearer picture of how and why chronic procrastination is linked to poor SRH. Our analyses also suggest that the link between chronic procrastination and SRH is relatively consistent across university student and adult samples and is unaffected by demographic factors.

**FIGURE 4:**
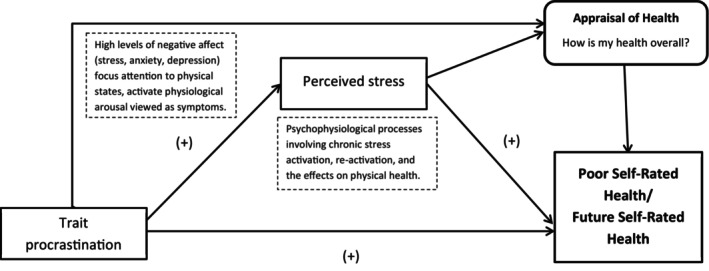
Integrated model of the roles of trait procrastination and stress in self‐rated health (SRH) as suggested by findings from the current study and by the procrastination‐health model (Sirois, 2007; Sirois et al., 2003), and the cognitive process model of self‐rated health (Jylhä, 2009), extended to future self‐rated health. Trait procrastination contributes to ratings of poor SRH both through psychophysiological pathways involving chronic stress activation and reactivation, and through the effects of high levels of negative affect on symptom awareness and perception. Stress as a downstream effect of procrastination can have both direct physiological effects on health status and impact symptom perception and awareness.

Another noteworthy contribution of the current research is the application of two models to understand not just how procrastination is linked to poor SRH, but also why. Consistent with both the cognitive process model (Jylhä, 2009) and the procrastination‐health model (Sirois, 2007; Sirois et al., 2003), we found evidence that higher perceived stress explains in part why procrastination is associated with poor SRH. This finding suggests that both models provide plausible and complementary explanations for why procrastination may be associated with poor SRH. However, in the absence of significant heterogeneity and therefore moderation via the demographic factors suggested by the cognitive process model, the procrastination‐health model may provide a more parsimonious explanation for why chronic procrastination contributes to poor SRH. Nonetheless, the consistency of our findings with both models suggests that the psychophysiological processes involved in repeated stress experiences linked to procrastinating, alongside the influence of stress on the cognitive processes involved in evaluating health states, provide reasonable explanations for the health risks associated with chronic procrastination. Figure 4 presents an integrated model of the role of stress in the procrastination–SRH link based on these two models and the current findings.

The current study also provided a novel test of the implications of chronic procrastination for expectations for future health states by testing a temporal variant of SRH, future SRH. Although the findings generally paralleled those for current SRH, there were several noteworthy differences. The magnitude of the average association with chronic procrastination was smaller for FSRH, and when adjusted for stress, the pooled association was no longer significant. There was also a significant degree of heterogeneity in the unadjusted effects that was due in part to participant sex, with the association between procrastination and poor FSRH becoming stronger for samples with a greater proportion of males. From the lens of the cognitive process model (Jylhä, 2009), biological sex is one personal‐historical factor that can shape evaluations of health in multiple ways, including through awareness of the gender gap in life expectancy, which favours women (Rochelle et al., 2015). Additionally, chronic procrastination is known to be more prevalent among males compared with females (Lu et al., 2022). Taken together, these and other factors may result in male procrastinators having more negative expectations for their future health states. With respect to the null findings for the moderator analysis of age in the procrastination–FSRH link, it is possible that the relatively young age of the majority of the samples (80.5% with a mean age between 20 and 35) reduced the amount of variance that could be explained by such an analysis.

The exploratory analysis of how trait procrastination might be implicated in the gap between evaluations of current and future SRH revealed interesting findings that highlight the role of biases in expectations for future health. Although procrastination was associated overall with poor future SRH, the small but significant pooled association with the discrepancy between SRH and future SRH indicates that for some samples, people prone to procrastination expected that their health in 10 years would be better than it was currently, even after controlling for age differences. This finding may seem counterintuitive given that procrastination was overall associated with expectations for poor health in the future. Indeed, in the current study, people, on average, tended to see their health remaining the same or worsening slightly over the next 10 years.

However, it is important to consider that this discrepancy analysis is relative to current health states. For example, current health may be viewed as poor by someone who chronically procrastinates; yet, they may believe that it will still be poor 10 years in the future but to a lesser degree than it is currently. Nonetheless, this expectation runs counter to what we know about the decline of health states in general over time, especially as a result of ageing (Jylhä, 2009). Accordingly, this optimistic bias towards future health among people who chronically procrastinate may be a reflection of wishful thinking (Sigall et al., 2000), and/or a general tendency to use avoidant coping to deal with unpleasant realizations (Sirois & Kitner, 2015). Thus, people prone to procrastination may avoid giving full consideration to their current poor health habits and states, including their stress levels, when appraising their future health. Alternatively, apparent optimistic views of health improving in the next 10 years may be fuelled in part by the difficulties in future‐oriented thinking associated with chronic procrastination (Blouin‐Hudon et al., 2016; Blouin‐Hudon & Pychyl, 2017; Sirois, 2014). Although this discrepancy was not very large, and for several samples there was no significant gap between current and future SRH, further research testing these competing explanations would be useful to provide more definitive insights into this unusual finding.

### Limitations and strengths

Though novel, the current findings should be considered in the context of several limitations. The current analyses relied upon data garnered from cross‐sectional studies, making it difficult to confirm the proposed directionality of the associations, which could be due to common method variance or conceptual overlap. Our analysis assumes the temporal precedence of trait procrastination in relation to SRH, based on research demonstrating that trait procrastination when measured by the GPS (Lay, 1986) has excellent temporal stability over a 10‐year period (Steel, 2007), and is considered a moderately heritable trait (Gustavson et al., 2014). Together, this evidence and the framework of the cognitive process model (Jylhä, 2009) provide support for expecting that procrastination influences SRH, rather than the reverse.

Because procrastination can be both a cause and consequence of stress (Johansson et al., 2023; Sirois, 2023; Sirois et al., 2023), determining the directionality of the links between procrastination and stress as suggested by the procrastination‐health model is less straightforward and cannot be established by the current analyses. We acknowledged this by focusing instead on the contributions of stress to explaining why trait procrastination might be linked to poor SRH. Consequently, further research examining the potentially dynamic, reciprocal, and likely mutually reinforcing linkages between procrastination and stress and their influence on evaluations of health is needed to confirm the predominant directionality.

It is possible that the behavioural route in the procrastination‐health model also explains the procrastination–SRH link. This was not investigated in the current study and could be a fruitful line of inquiry for future research to explore.

The strengths of the current research include testing our research hypotheses across large sets of independent and diverse samples, with the overall statistical meta‐analysis of procrastination and SRH conducted across 36 samples totalling 8603 participants. This MOD analytic approach provided support for the generalisability of the findings across student and non‐student samples, and the opportunity to conduct robust tests of how and why procrastination is linked to poor SRH to build a strong evidence base for an understudied area (Cumming, 2014). This would not have been possible with a traditional meta‐analysis framework which requires having a sufficient evidence base to analyse.

### Conclusions

This analysis of 36 samples provides robust evidence that chronic procrastination is associated with poor self‐rated current and, to a lesser extent, FSRH. Consistent with the cognitive process model (Jylhä, 2009) and the procrastination‐health model (Sirois, 2007; Sirois et al., 2003), we found evidence that high levels of perceived stress contribute in part to why people prone to procrastination report poorer overall health. Exploratory analyses suggest that some people prone to procrastination may hold unrealistically optimistic expectations about their future health, which could undermine motivation to adopt healthier behaviours. Taken together, these findings underscore the importance of considering chronic procrastination not only as self‐regulatory failure with academic or occupational costs but also as a behaviour pattern with significant negative implications for overall health status.

## AUTHOR CONTRIBUTIONS

**Fuschia M. Sirois:** Conceptualization; investigation; writing – original draft; methodology; writing – review and editing; formal analysis; project administration; data curation; resources. **Cormac Monaghan:** Conceptualization; writing – original draft; methodology; visualization; writing – review and editing; formal analysis; data curation.

## CONFLICT OF INTEREST STATEMENT

The authors declare no conflicts of interest.

## Supporting information


**Figure S1.** Distribution of discrepancy scores (self‐rated health: SRH – future FSRH) across 20 samples. Note that SRH and FSRH were coded with higher values reflecting poorer health.

**Figure S2.** Forest plot of study‐specific and pooled meta‐analytic estimates (*β*, 95% CI) for the link between procrastination and self‐rated health discrepancies (after controlling for age). Note that SRH and FSRH were coded with higher values reflecting poorer health.

## Data Availability

All analysis code is publicly available at https://github.com/C‐Monaghan/marigold‐mailbox and https://doi.org/10.17605/OSF.IO/3APFG. Data is available by reasonable request from the authors, and where allowable.

## References

[bjhp70099-bib-0001] Barry, L. E. , O'Neill, S. , Heaney, L. G. , & O'Neill, C. (2021). Stress‐related health depreciation: Using allostatic load to predict self‐rated health. Social Science & Medicine, 283, 114170. 10.1016/j.socscimed.2021.114170 34216886

[bjhp70099-bib-0002] Basirimoghadam, M. , Rafii, F. , & Ebadi, A. (2020). Self‐rated health and general procrastination in nurses: A cross‐sectional study. Pan African Medical Journal, 36, 254. 10.11604/pamj.2020.36.254.23720 33014250 PMC7519796

[bjhp70099-bib-0003] Blouin‐Hudon, E.‐M. C. , & Pychyl, T. A. (2017). A mental imagery intervention to increase future self‐continuity and reduce procrastination. Applied Psychology, 66(2), 326–352. 10.1111/apps.12088

[bjhp70099-bib-0004] Blouin‐Hudon, E.‐M. C. , Sirois, F. M. , & Pychyl, T. A. (2016). Temporal views of procrastination, health and well‐being. In F. M. Sirois & T. Pychyl (Eds.), Procrastination, health, and well‐being (pp. 213–232). Academic Press. 10.1016/B978-0-12-802862-9.00008-6

[bjhp70099-bib-0005] Borenstein, M. , Hedges, L. , Higgins, J. , & Rothstein, H. (2022). Comprehensive meta‐analysis version 4. Biostat.

[bjhp70099-bib-0006] Card, N. A. (2012). Applied meta‐analysis for social science research. Guilford Press.

[bjhp70099-bib-0007] Cohen, J. (1988). Statistical power analysis for the behavioral sciences (2nd ed.). Erlbaum.

[bjhp70099-bib-0008] Cohen, S. , Janicki‐Deverts, D. , Doyle, W. J. , Miller, G. E. , Frank, E. , Rabin, B. S. , & Turner, R. B. (2012). Chronic stress, glucocorticoid receptor resistance, inflammation, and disease risk. Proceedings of the National Academy of Sciences, 109(16), 5995–5999. 10.1073/pnas.1118355109 PMC3341031 22474371

[bjhp70099-bib-0009] Cohen, S. , Kamarck, T. , & Mermelstein, R. (1983). A global measure of perceived stress. Journal of Health and Social Behavior, 24(4), 385–396. 6668417

[bjhp70099-bib-0010] Cohen, S. , & Williamson, G. M. (1991). Stress and infectious disease in humans. Psychological Bulletin, 109, 5–24. 2006229 10.1037/0033-2909.109.1.5

[bjhp70099-bib-0011] Cumming, G. (2014). The new statistics: Why and how. Psychological Science, 25(1), 7–29. 10.1177/0956797613504966 24220629

[bjhp70099-bib-0012] Ferrari, J. R. , O'Callaghan, J. , & Newbegin, I. (2005). Prevalence of procrastination in the United States, United Kingdom, and Australia: Arousal and avoidance delays in adults. North American Journal of Psychology, 7, 1–6.

[bjhp70099-bib-0013] Fischer, S. , Nater, U. M. , & Laferton, J. A. C. (2016). Negative stress beliefs predict somatic symptoms in students under academic stress. International Journal of Behavioral Medicine, 23(6), 746–751. 10.1007/s12529-016-9562-y 27090420 PMC5118394

[bjhp70099-bib-0014] Flett, G. L. , Stainton, M. , Hewitt, P. , Sherry, S. , & Lay, C. (2012). Procrastination automatic thoughts as a personality construct: An analysis of the procrastinatory cognitions inventory. Journal of Rational‐Emotive & Cognitive‐Behavior Therapy, 30, 223–236. 10.1007/s10942-012-0150-z

[bjhp70099-bib-0015] Gustavson, D. E. , Miyake, A. , Hewitt, J. K. , & Friedman, N. P. (2014). Genetic relations among procrastination, impulsivity, and goal‐management ability: Implications for the evolutionary origin of procrastination. Psychological Science, 25, 1178–1188. 24705635 10.1177/0956797614526260 PMC4185275

[bjhp70099-bib-0016] Hen, M. , & Goroshit, M. (2018). General and life‐domain procrastination in highly educated adults in Israel. Frontiers in Psychology, 9, 1173. 10.3389/fpsyg.2018.01173 30022965 PMC6039828

[bjhp70099-bib-0017] Hirotsu, C. , Tufik, S. , & Andersen, M. L. (2015). Interactions between sleep, stress, and metabolism: From physiological to pathological conditions. Sleep Science, 8(3), 143–152. 10.1016/j.slsci.2015.09.002 26779321 PMC4688585

[bjhp70099-bib-0018] Jakobsen, J. C. , Gluud, C. , Wetterslev, J. , & Winkel, P. (2017). When and how should multiple imputation be used for handling missing data in randomised clinical trials – A practical guide with flowcharts. BMC Medical Research Methodology, 17(1), 162. 10.1186/s12874-017-0442-1 29207961 PMC5717805

[bjhp70099-bib-0019] Johansson, F. , Rozental, A. , Edlund, K. , Côté, P. , Sundberg, T. , Onell, C. , Rudman, A. , & Skillgate, E. (2023). Associations between procrastination and subsequent health outcomes among university students in Sweden. JAMA Network Open, 6(1), e2249346. 10.1001/jamanetworkopen.2022.49346 36598789 PMC9857662

[bjhp70099-bib-0020] Jylhä, M. (2009). What is self‐rated health and why does it predict mortality? Towards a unified conceptual model. Social Science & Medicine, 69(3), 307–316. 19520474 10.1016/j.socscimed.2009.05.013

[bjhp70099-bib-0021] Kananen, L. , Enroth, L. , Raitanen, J. , Jylhävä, J. , Bürkle, A. , Moreno‐Villanueva, M. , Bernhardt, J. , Toussaint, O. , Grubeck‐Loebenstein, B. , Malavolta, M. , Basso, A. , Piacenza, F. , Collino, S. , Gonos, E. S. , Sikora, E. , Gradinaru, D. , Jansen, E. , Dollé, M. E. T. , Salmon, M. , … Jylhä, M. (2021). Self‐rated health in individuals with and without disease is associated with multiple biomarkers representing multiple biological domains. Scientific Reports, 11(1), 6139. 10.1038/s41598-021-85668-7 33731775 PMC7969614

[bjhp70099-bib-0022] Kelly, S. M. , & Walton, H. R. (2021). “I'll work out tomorrow”: The procrastination in exercise scale. Journal of Health Psychology, 26(13), 2613–2625. 10.1177/1359105320916541 32459106

[bjhp70099-bib-0023] Klingsieck, K. B. (2013). Procrastination: When good things don't come to those who wait. European Psychologist, 18(1), 24–34. 10.1027/1016-9040/a000138

[bjhp70099-bib-0024] Lay, C. H. (1986). At last, my research article on procrastination. Journal of Research in Personality, 20, 474–495. 10.1016/0092-6566(86)90127-3

[bjhp70099-bib-0025] Lee, E.‐H. (2012). Review of the psychometric evidence of the perceived stress scale. Asian Nursing Research, 6(4), 121–127. 10.1016/j.anr.2012.08.004 25031113

[bjhp70099-bib-0026] Li, X. , Buxton, O. M. , Kim, Y. , Haneuse, S. , & Kawachi, I. (2020). Do procrastinators get worse sleep? Cross‐sectional study of US adolescents and young adults. SSM ‐ Population Health, 10, 100518. 10.1016/j.ssmph.2019.100518 31799365 PMC6881694

[bjhp70099-bib-0027] Little, R. J. A. (1988). A test of missing completely at random for multivariate data with missing values. Journal of the American Statistical Association, 83(404), 1198–1202. 10.1080/01621459.1988.10478722

[bjhp70099-bib-0028] Löckenhoff, C. E. , Terracciano, A. , Ferrucci, L. , & Costa, P. T. (2012). Five‐factor personality traits and age trajectories of self‐rated health: The role of question framing. Journal of Personality, 80(2), 375–401. 21299558 10.1111/j.1467-6494.2011.00724.x PMC3248623

[bjhp70099-bib-0029] Lu, D. , He, Y. , & Tan, Y. (2022). Gender, socioeconomic status, cultural differences, education, family size and procrastination: A sociodemographic meta‐analysis. Frontiers in Psychology, 12, 719425. 10.3389/fpsyg.2021.719425 35069309 PMC8766341

[bjhp70099-bib-0030] Manap, A. , Rizzo, A. , Yıldırmaz, A. , Dilekçi, Ü. , & Yıldırım, M. (2024). The mediating role of procrastination in the relationship between fear of missing out and internet addiction in university students. International Journal of Environmental Research and Public Health, 21(1), 49. 10.3390/ijerph21010049 PMC10815717 38248514

[bjhp70099-bib-0031] McCown, W. G. , & Johnson, J. L. (2001). The adult inventory of procrastination revised.

[bjhp70099-bib-0032] Reinecke, L. , Meier, A. , Beutel, M. E. , Schemer, C. , Stark, B. , Wölfling, K. , & Müller, K. W. (2018). The relationship between trait procrastination, internet use, and psychological functioning: Results from a community sample of German adolescents. Frontiers in Psychology, 9, 9139. 10.3389/fpsyg.2018.00913 PMC6004405 29942268

[bjhp70099-bib-0033] Rochelle, T. L. , Yeung, D. K. Y. , Bond, M. H. , & Li, L. M. W. (2015). Predictors of the gender gap in life expectancy across 54 nations. Psychology, Health & Medicine, 20(2), 129–138. 10.1080/13548506.2014.936884 25005485

[bjhp70099-bib-0034] Rosenthal, R. (1979). The file drawer problem and tolerance for null results. Psychological Bulletin, 86, 638–641. 10.1037/0033-2909.86.3.638

[bjhp70099-bib-0035] Shooshtari, S. , Menec, V. , & Tate, R. (2007). Comparing predictors of positive and negative self‐rated health between younger (25–54) and older (55+) Canadian adults: A longitudinal study of well‐being. Research on Aging, 29(6), 512–554. 10.1177/0164027507305729

[bjhp70099-bib-0036] Sigall, H. , Kruglanski, A. , & Fyock, J. (2000). Wishful thinking and procrastination. Journal of Social Behavior and Personality, 15(5), 283–296.

[bjhp70099-bib-0037] Sirois, F. M. (2007). “I'll look after my health, later”: A replication and extension of the procrastination–health model with community‐dwelling adults. Personality and Individual Differences, 43, 15–26. 10.1016/j.paid.2006.11.003

[bjhp70099-bib-0038] Sirois, F. M. (2014). Out of sight, out of time? A meta‐analytic investigation of procrastination and time perspective. European Journal of Personality, 28, 511–520. 10.1002/per.1947

[bjhp70099-bib-0039] Sirois, F. M. (2015a). Is procrastination a vulnerability factor for hypertension and cardiovascular disease? Testing an extension of the procrastination‐health model. Journal of Behavioral Medicine, 38, 578–589. 25804373 10.1007/s10865-015-9629-2

[bjhp70099-bib-0040] Sirois, F. M. (2015b). Who looks forward to better health? Personality factors and future self‐rated health in the context of chronic illness. International Journal of Behavioral Medicine, 22, 569–579. 10.1007/s12529-015-9460-8 25622814

[bjhp70099-bib-0041] Sirois, F. M. (2021). Trait procrastination undermines outcome and efficacy expectancies for achieving health‐related possible selves. Current Psychology, 40(8), 3840–3847. 10.1007/s12144-019-00338-2 34776720 PMC8550689

[bjhp70099-bib-0042] Sirois, F. M. (2023). Procrastination and stress: A conceptual review of why context matters. International Journal of Environmental Research and Public Health, 20(6), 5031. 36981941 10.3390/ijerph20065031 PMC10049005

[bjhp70099-bib-0043] Sirois, F. M. , & Biskas, M. (2024). Procrastination and health in nurses: Investigating the roles of stress, health Behaviours and social support. International Journal of Environmental Research and Public Health, 21(7), 898. 39063475 10.3390/ijerph21070898 PMC11277167

[bjhp70099-bib-0044] Sirois, F. M. , & Kitner, R. (2015). Less adaptive or more maladaptive? A meta‐analytic investigation of procrastination and coping. European Journal of Personality, 29, 433–444. 10.1002/per.1985

[bjhp70099-bib-0045] Sirois, F. M. , Melia‐Gordon, M. L. , & Pychyl, T. A. (2003). “I'll look after my health, later”: An investigation of procrastination and health. Personality and Individual Differences, 35(5), 1167–1184. 10.1016/S0191-8869(02)00326-4

[bjhp70099-bib-0046] Sirois, F. M. , & Molnar, D. S. (2017). Perfectionistic strivings and concerns are differentially associated with self‐rated health beyond negative affect. Journal of Research in Personality, 70, 73–83. 10.1016/j.jrp.2017.06.003

[bjhp70099-bib-0047] Sirois, F. M. , Molnar, D. S. , & Hirsch, J. K. (2017). A meta‐analytic and conceptual update on the associations between procrastination and multidimensional perfectionism. European Journal of Personality, 31(2), 137–159. 10.1002/per.2098

[bjhp70099-bib-0048] Sirois, F. M. , & Pychyl, T. A. (2013). Procrastination and the priority of short‐term mood regulation: Consequences for future self. Social and Personality Psychology Compass, 7(2), 115–127. 10.1111/spc3.12011

[bjhp70099-bib-0049] Sirois, F. M. , Stride, C. B. , & Pychyl, T. A. (2023). Procrastination and health: A longitudinal test of the roles of stress and health behaviours. British Journal of Health Psychology, 28(3), 860–875. 10.1111/bjhp.12658 36919887

[bjhp70099-bib-0050] Sirois, F. M. , Van Eerde, W. , & Argiropoulou, M. I. (2015). Is procrastination related to sleep quality? Testing an extension of the procrastination‐health model. Cogent Psychology, 2(1). 10.1080/23311908.2015.1074776

[bjhp70099-bib-0051] Sirois, F. M. , Yang, S. , & van Eerde, W. (2019). Development and validation of the general procrastination scale (GPS‐9): A short and reliable measure of trait procrastination. Personality and Individual Differences, 146, 26–33. 10.1016/j.paid.2019.03.039

[bjhp70099-bib-0052] Smyth, J. , Zawadzki, M. , & Gerin, W. (2013). Stress and disease: A structural and functional analysis. Social and Personality Psychology Compass, 7(4), 217–227. 10.1111/spc3.12020

[bjhp70099-bib-0053] Stainton, M. , Lay, C. H. , & Flett, G. L. (2000). Trait procrastinators and behavior/trait‐specific cognitions. Journal of Social Behavior and Personality, 15(5), 297–312.

[bjhp70099-bib-0054] Steel, P. (2007). The nature of procrastination: A meta‐analytic and theoretical review of quintessential self‐regulatory failure. Psychological Bulletin, 133(1), 65–94. 10.1037/0033-2909.133.1.65 17201571

[bjhp70099-bib-0055] Svedberg, P. , Bardage, C. , Sandin, S. , & Pedersen, N. L. (2006). A prospective study of health, life‐style and psychosocial predictors of self‐rated health. European Journal of Epidemiology, 21(10), 767–776. 10.1007/s10654-006-9064-3 17106761

[bjhp70099-bib-0056] Team, R. C. (2025). R: A language and environment for statistical computing. R Foundation for Statistical Computing. https://www.R‐project.org/

[bjhp70099-bib-0057] Tierney, N. , & Cook, D. (2023). Expanding tidy data principles to facilitate missing data exploration, visualization and assessment of imputations. Journal of Statistical Software, 105(7), 1–31. 10.18637/jss.v105.i07 36798141

[bjhp70099-bib-0058] van Buuren, S. , & Groothuis‐Oudshoorn, K. (2011). Mice: Multivariate imputation by chained equations in R. Journal of Statistical Software, 45(3), 1–67. 10.18637/jss.v045.i03

[bjhp70099-bib-0059] van Eerde, W. (2003). A meta‐analytically derived nomological network of procrastination. Personality and Individual Differences, 35(6), 1401–1418. 10.1016/S0191-8869(02)00358-6

[bjhp70099-bib-0060] Viechtbauer, W. (2010). Conducting meta‐analyses in R with the metafor package. Journal of Statistical Software, 36(3), 1–48. 10.18637/jss.v036.i03

[bjhp70099-bib-0061] Ware, J. E. J. , & Sherbourne, C. D. (1992). The MOS 36‐item short‐form health survey (SF‐36): I. Conceptual framework and item selection. Medical Care, 30(6), 473–483. 1593914

[bjhp70099-bib-0062] Wirtz, P. H. , & von Känel, R. (2017). Psychological stress, inflammation, and coronary heart disease. Current Cardiology Reports, 19(11), 111. 10.1007/s11886-017-0919-x 28932967

[bjhp70099-bib-0063] Zeileis, A. , & Grothendieck, G. (2005). Zoo: S3 infrastructure for regular and irregular time series. Journal of Statistical Software, 14(6), 1–27. 10.18637/jss.v014.i06

