# Supplementary material for: Procrastination, stress, and self‐rated health: A multi‐sample meta‐analysis
Source: Br J Health Psychol. 2026 Aug 8;31(3):e70099. doi: 10.1111/bjhp.70099 (PMC13452009; doi:10.1111/bjhp.70099)
Supplement: Supplementary file 1 [file BJHP-31-0-s001.docx]

**Supplement Figure 2.** Forest plot of study-specific and pooled meta-analytic estimates ($\beta$, 95% CI) for the link between procrastination and self-rated health discrepancies (after controlling for age). Note that SRH and FSRH were coded with higher values reflecting poorer health.
