# Supplementary material for: Procrastination, stress, and self‐rated health: A multi‐sample meta‐analysis
Source: Br J Health Psychol. 2026 Aug 8;31(3):e70099. doi: 10.1111/bjhp.70099 (PMC13452009; doi:10.1111/bjhp.70099)
Supplement: Supplementary file 2 [file BJHP-31-0-s002.docx]

**Supplement Figure 1.** Distribution of discrepancy scores (self-rated health: SRH – future FSRH) across 20 samples. Note that SRH and FSRH were coded with higher values reflecting poorer health.
